# A Longitudinal Epidemiology Study of Meningococcal Carriage in Students 13 to 25 Years Old in Quebec

**DOI:** 10.1128/mSphere.00427-18

**Published:** 2018-12-05

**Authors:** Rodica Gilca, Philippe De Wals, Sheila M. Nolan, Nicholas Kitchin, Joseph J. Eiden, Qin Jiang, C. Hal Jones, Kathrin U. Jansen, Annaliesa S. Anderson, Louise Pedneault

**Affiliations:** a Research Center, Centre Hospitalier Universitaire de Québec-Université Laval, Québec City, Quebec, Canada; b Department of Social and Preventive Medicine, Laval University, Québec City, Quebec, Canada; c Vaccine Clinical Research and Development, Pfizer Inc., Pearl River, New York, USA; d Vaccine Clinical Research and Development, Pfizer Ltd., Hurley, Berkshire, United Kingdom; e Vaccine Clinical Research and Development, Pfizer Inc., Collegeville, Pennsylvania, USA; f Vaccine Research and Development, Pfizer Inc., Pearl River, New York, USA; Antimicrobial Development Specialists, LLC

**Keywords:** *Neisseria meningitidis*, NmB, epidemiology, factor H binding protein, oropharyngeal carriage, serogroup B, vaccines

## Abstract

Neisseria meningitidis carriage data are necessary to inform serogroup B (NmB) immunization program implementation. This longitudinal study compared detection methods to measure N. meningitidis throat carriage prevalence in Quebec from November 2010 to December 2013 using cultured swab isolates and direct swab PCR from students in ninth grade (aged 13 to 15 years; *n* = 534) and eleventh grade/college entry (16 to 18 years; *n* = 363) and in university students in dormitories (18 to 25 years; *n* = 360) at 3 time points per group. Meningococcal and NmB carriage rates were lower in ninth- and eleventh-grade/college entry students than university students, regardless of methodology. Genotyping cultured isolates by PCR detected NmB and non-NmB in 2.1% and 7.3% of ninth-grade students, in 1.7% and 7.2% of eleventh-grade/college entry students, and in 7.5% and 21.9% of university students, respectively. NmB acquisition rates were 1.9, 0.7, and 3.3 per 1,000 person-months across respective age groups. Most NmB isolates (94.7%, 76.9%, and 86.8%, respectively) expressed subfamily A factor H binding-protein (fHBP) variants. The most common non-NmB serogroups were NmY (1.7%/1.1%) from ninth grade and eleventh grade/college entry and NmW (2.8%) from university students. Genomic analyses detected disease-associated sequence types in carriage isolates, and carriage could persist for months. This is the largest longitudinal carriage study in Canada and the first to report fHBP variants in NmB carriage isolates in healthy Canadians. These data contribute to identification of the optimal window for NmB vaccination in precollege adolescents and provide a baseline for investigating NmB vaccination effects on carriage in this population.

**IMPORTANCE** Disease caused by Neisseria meningitidis is associated with serious complications and a high fatality rate. Asymptomatic individuals can harbor the bacterium in the throat, a state known as “carriage,” which can lead to person-to-person spread of the pathogen. This study examined N. meningitidis carriage from 2010 to 2013 among 2 groups in the Quebec City region: ninth-grade students (aged 13 to 15 years), who were also followed in their last year of high school (eleventh grade/college entry; 16 to 18 years), and university students (18 to 25 years); both groups have been shown in some other geographic regions to have high rates of carriage. This study demonstrated that N. meningitidis carriage rates were higher among university students in dormitories than ninth-grade and eleventh-grade/college entry students. Understanding carriage rates in these age groups leads to better strategies to control N. meningitidis by targeting vaccination to those responsible for transmission within the population.

## Abstract (precis)

Disease caused by Neisseria meningitidis is associated with serious complications and a high fatality rate. Asymptomatic individuals can harbor the bacterium in the throat, a state known as “carriage,” which can lead to person-to-person spread of the pathogen. This study examined N. meningitidis carriage from 2010 to 2013 among 2 groups in the Quebec City region: ninth-grade students (aged 13 to 15 years), who were also followed in their last year of high school (eleventh grade/college entry; 16 to 18 years), and university students (18 to 25 years); both groups have been shown in some other geographic regions to have high rates of carriage. This study demonstrated that N. meningitidis carriage rates were higher among university students in dormitories than ninth-grade and eleventh-grade/college entry students. Understanding carriage rates in these age groups leads to better strategies to control N. meningitidis by targeting vaccination to those responsible for transmission within the population.

## INTRODUCTION

The pathogenic bacterium Neisseria meningitidis causes invasive meningococcal disease (IMD). IMD is generally highest in infants <1 year of age and adolescents/young adults 15 to 24 years of age (1). Most IMD cases are caused by serogroups NmA, NmB, NmC, NmW, NmX, and NmY (1). Between 1997 and 2011 in Quebec province, serogroups NmB, NmC, NmY, and NmW accounted for 68%, 20%, 8%, and 3% of IMD cases, respectively (2). In Canada, Quebec has one of the highest rates of IMD caused by N. meningitidis serogroup B (NmB) due to the emergence of a virulent ST-269 clone in 2003 (3, 4).

N. meningitidis is commonly associated with asymptomatic throat carriage, the prevalence of which varies with age and living conditions (5). In European and other countries where NmB and NmC predominate, carriage rates in the absence of vaccination increase gradually through early childhood, then increase substantially between ages 15 and 19 years, before decreasing and stabilizing at <10% during early adulthood (6). Carriage in the mid-to-late teen age group is implicated as the reservoir for disease; this is supported by data from NmC vaccination campaigns and protection against invasive disease that extended to age groups that did not receive vaccine (7–9).

In Quebec, mass immunization campaigns against NmC were triggered by NmC outbreaks and targeted those aged 6 months to 20 years in 1992 to 1993 (polysaccharide vaccine) and aged 2 months to 20 years in 2001 (mainly conjugate vaccine) (10, 11). One dose of NmC conjugate vaccine at age 12 months was introduced into the routine Quebec immunization program in 2002; an adolescent booster dose was added in 2013. The NmACWY conjugate vaccine has been offered in Canada since 2006 to individuals at high risk for IMD (12). The proportion of IMD cases in Quebec caused by NmC decreased from 61% to 1% from 2001 to 2011, whereas the proportion of IMD cases due to NmB increased from 29% to 88% (2).

Two vaccines are licensed for prevention of NmB IMD. MenB-FHbp (Trumenba, Bivalent rLP2086; Pfizer, Philadelphia, PA), an NmB vaccine containing 2 recombinant NmB factor H binding protein (fHBP) variants, 1 each from subfamilies A and B, was approved in the United States in October 2014 and in Canada in October 2017. MenB-4C (Bexsero, 4CMenB; GlaxoSmithKline Vaccines, Srl, Siena, Italy), composed of recombinant neisserial adhesin A (NadA), neisserial heparin binding antigen (NHBA), fHBP (subfamily B, variant 1), and PorA variant P1.7-2,4 expressed in outer membrane vesicles, was approved in Canada in December 2013 and in the United States in January 2015. In Quebec, MenB-4C is recommended for high-risk groups; an immunization campaign conducted in 2014 aimed to control increased NmB IMD in the Saguenay-Lac-Saint-Jean region north of Quebec City (13).

This study provides epidemiological data on meningococcal carriage in the Quebec City region for 2 cohorts during 2010 to 2013 (before licensure of NmB vaccines in Canada): ninth-grade students (aged 13 to 15 years), who were followed up in their last year of high school (eleventh grade/college entry; aged 16 to 18 years), and university students (aged 18 to 25 years). Data presented provide useful information for informing strategies to control NmB IMD through vaccination of the age group responsible for transmission of virulent clones in the population. Additionally, this study provided an opportunity to compare different methodological approaches for detection and capsular grouping of N. meningitidis carriage isolates and provided a perspective on circulating meningococcal strains in the region.

## RESULTS

### Subject disposition.

**FIG 1:**
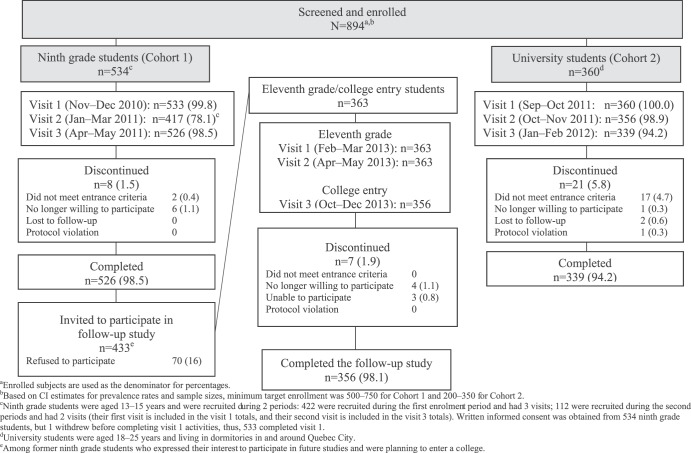
Disposition of subjects (intent-to-treat population).

A total of 894 subjects were screened and enrolled in the initial study, of whom 534 were ninth-grade students (cohort 1; median age [range], 14.0 years [13 to 15 years]) and 360 were university students (cohort 2; median age [range], 19.0 years [18 to 25 years]) (Fig. 1). Among ninth-grade students, 422 enrolled at visit 1 and an additional 112 enrolled during the visit 2 interval. Former ninth-grade students (*n* = 433) were invited to participate in the follow-up study in eleventh grade (cohort 1 follow-up); 363 were enrolled (median age [range], 16.0 years [16 to 18 years]). Overall, 526 ninth-grade students and 339 university students completed the initial study, and 356 of 363 eleventh-grade/college entry students completed the follow-up portion of the study. Age differences notwithstanding, demographic and clinical characteristics were generally similar between cohorts 1 and 2, with 3% to 5% of subjects recorded as nonwhite and slightly more than half being female. Most participants (>90%) had received a serogroup C conjugate vaccine during the 2001 immunization campaign; between 1.5% and 3.4% at visits 1 to 3 received antibiotics within the previous 2 weeks.

### NmB carriage.

**FIG 2:**
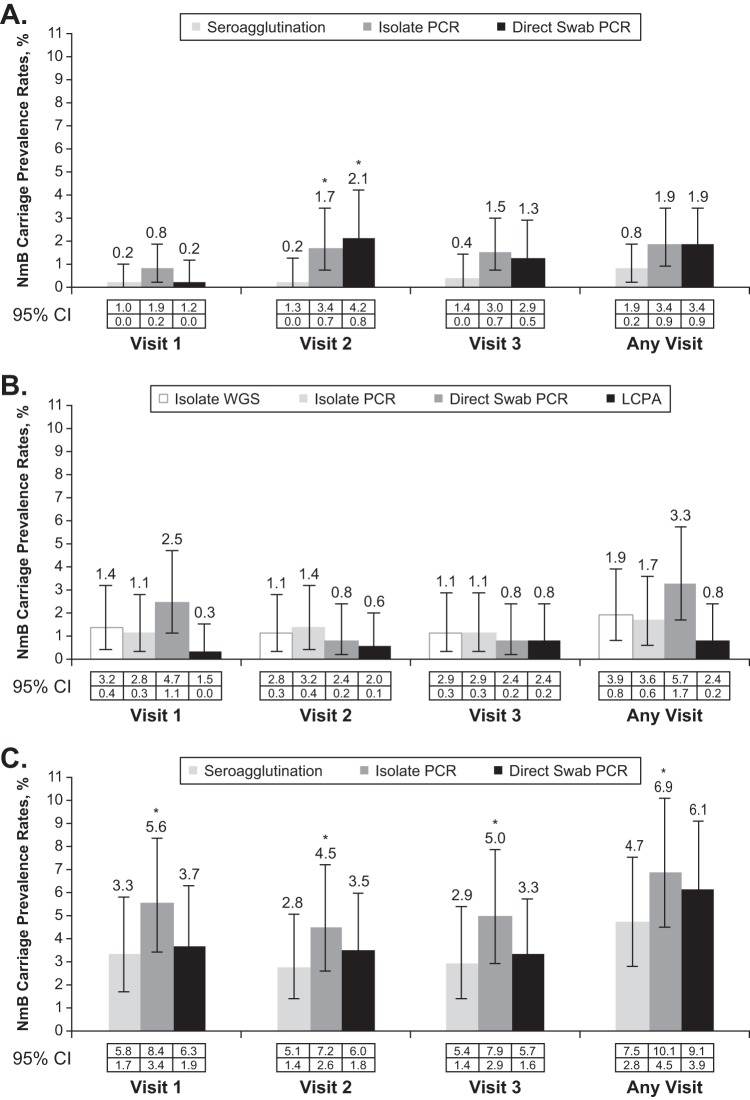
Neisseria meningitidis serogroup B (NmB) carriage prevalence rates at each visit for both cohorts. NmB carriage was determined by seroagglutination, isolate PCR, and direct swab PCR assays at 3 visits and any visit for ninth-grade students (A) and university students (C) and by isolate whole-genome sequencing (WGS), isolate PCR, direct swab PCR, and live cell phenotypic assay (LCPA) for eleventh-grade/college entry students (B). *, *P < *0.05. CIs were calculated using the exact method based on Clopper-Pearson (2-sided). The McNemar test using the exact method was used to compare the prevalence rates between PCR analyses and seroagglutination for each visit in ninth-grade/university students.

Regardless of methodology, NmB carriage rates were higher in university students at all visits. NmB carriage rates determined by isolate PCR analyses were 1.9% in ninth-grade students, 1.7% among cohort 1 follow-up subjects at eleventh grade/college entry, and 6.9% in university students at any visit (Fig. 2).

During the study period, 1.1% (6/529) of ninth-grade students, 0.6% (2/359) of eleventh-grade/college entry students, and 2.0% (7/345) of university students who tested negative for NmB at visit 1 or 2 acquired NmB, and 25.0% (2/8), 40% (2/5), and 38.1% (8/21), respectively, of those who were NmB carriers at visit 1 or 2 became negative for NmB carriage at subsequent visits. A small proportion (0.8% [3/356]) of eleventh-grade/college entry students who were negative in ninth grade became NmB carriers at the time of the follow-up study 2 years later. Among 7 students who were carriers at visit 1 or 2, 6 (86%) did not have NmB detected during subsequent visits.

The NmB acquisition rate at visits 1 to 3 per 1,000 person-months was 1.9 for ninth-grade students, 0.7 for eleventh-grade/college entry students, and 3.3 for university students. Only 1.9% (10/533) of ninth-grade students and 1.9% (7/363) of eleventh-grade/college entry students compared with 6.9% (25/360) of university students were NmB carriers at any time during the study, of whom 7, 5, and 17, respectively, were carriers at >1 visit. Seven, 5, and 15 students, respectively, were carriers at ≥2 consecutive visits.

### Non-NmB meningococcal carriage.

Carriage prevalence rates by isolate PCR for all non-NmB meningococci (NmA, NmC, NmE, NmW, NmX, NmY, NmZ, and nongroupable) in ninth-grade and eleventh-grade/college entry students were approximately one-third the rates in university students at any visit (7.3% [39/533] and 7.2% [26/363] versus 21.9% [79/360]) and for each individual visit (Table 1). The most frequently detected non-NmB groups in ninth-grade, eleventh-grade/college entry, and university students were NmY, with rates at any visit of approximately 1.1% to 2.2% in each age group, and NmW, with rates of 0.3% to 0.9% in ninth-grade and eleventh-grade/college entry students and 2.5% in university students. Prevalence of NmC was very low in all age groups; no NmA or NmX isolates were detected (Table 1). Among NmC carriers, 8 subjects were carriers at ≥1 visit. All 8 had previously received N. meningitidis vaccination: 6 received NmC conjugate vaccine, 1 received N. meningitidis polysaccharide vaccine, and 1 received an unknown N. meningitidis vaccine.

**TABLE 1:** Summary of meningococcal carriage prevalence rates at each visit by isolate PCR (ITT population)^*g*^

Visit (swab)	Cohort	Age group (*N*^*e*^)	*N. meningitidis* serogroup detection from throat swab, *n* (%)^*a*^^,^^*b*^							
			NmB^*c*^	NmC	NmY	NmW	Nongroupable	NmE or NmZ	All non-NmB^*d*^	All meningococci
Visit 1	1	9th grade (533)	4 (0.8)	2 (0.4)	8 (1.5)	4 (0.8)	16 (3.0)	3 (0.6)	33 (6.2)	37 (6.9)
		11th grade^*f*^ (363)	4 (1.1)	1 (0.3)	3 (0.8)	1 (0.3)	10 (2.8)	2 (0.6)	17 (4.7)	21 (5.8)
	2	University (360)	20 (5.6)	3 (0.8)	5 (1.4)	8 (2.2)	35 (9.7)	16 (4.4)	67 (18.6)	87 (24.2)
Visit 2	1	9th grade (417)	7 (1.7)	1 (0.2)	4 (1.0)	1 (0.2)	15 (3.6)	0 (0.0)	21 (5.0)	28 (6.7)
		11th grade (363)	5 (1.4)	1 (0.3)	2 (0.6)	0 (0.0)	8 (2.2)	2 (0.6)	13 (3.6)	18 (5.0)
	2	University (356)	16 (4.5)	3 (0.8)	3 (0.8)	6 (1.7)	31 (8.7)	9 (2.5)	52 (14.6)	68 (19.1)
Visit 3	1	9th grade (526)	8 (1.5)	1 (0.2)	5 (1.0)	3 (0.6)	14 (2.7)	1 (0.2)	24 (4.6)	32 (6.1)
		College entry (356)	4 (1.1)	0 (0.0)	2 (0.6)	1 (0.3)	12 (3.4)	3 (0.8)	18 (5.1)	22 (6.2)
	2	University (339)	17 (5.0)	4 (1.2)	6 (1.8)	7 (2.1)	27 (8.0)	8 (2.4)	52 (15.3)	69 (20.4)
Any visit	1	9th grade (533)	11 (2.1)	2 (0.4)	9 (1.7)	5 (0.9)	19 (3.6)	3 (0.6)	39 (7.3)	49 (9.2)
		11th grade/college entry (363)	6 (1.7)	1 (0.3)	4 (1.1)	1 (0.3)	17 (4.7)	3 (0.8)	26 (7.2)	32 (8.8)
	2	University (360)	27 (7.5)	4 (1.1)	8 (2.2)	9 (2.5)	42 (11.7)	15 (4.2)	79 (21.9)	105 (29.1)

a *n* (%) is the number and percentage of subjects with positive meningococci carriage at that visit.

b No NmA or NmX isolates were detected.

c NmB PCR data were confirmed by WGS.

d All non-NmB meningococci: A, C, X, Y, W, nongroupable, Z, and E.

e *N* is number of subjects who had a culture performed at that visit.

f Eleventh-grade/college-entry students are the same (former) ninth-grade students.

g Abbreviations: ITT, intent-to-treat; Nm, *Neisseria meningitidis*; isolate PCR assay was performed by real-time PCR. Non-NmB meningococci include any sample in which the *porA* and/or *ctrA* gene(s) was detected but the group B capsule gene was not detected. Missing data were not imputed.

During the study period, 2.0% (10/507) of ninth-grade students, 3.1% (11/357) of eleventh-grade/college entry students, and 5.8% (18/313) of university students who were negative for all non-NmB meningococci carriage at visit 1 or 2 became carriers, and 51.4% (19/37), 52.9% (9/17), and 43.1% (31/72), respectively, of those who were carriers at visit 1 or 2 became negative for carriage.

Acquisition rates at visits 1 to 3 for non-NmB isolates were 0.7 per 1,000 person-months for both ninth-grade and eleventh-grade/college entry students and 1.0 per 1,000 person-weeks for university students. Acquisition rates for nongroupable isolates were 1.6 and 3.6 per 1,000 person-months for ninth-grade and eleventh-grade/college entry students, respectively, and 1.5 per 1,000 person-weeks for university students.

### All meningococcal carriage.

Overall carriage prevalence by isolate PCR remained relatively consistent across time points within cohorts (Table 1). Prevalence across visits ranged from 6.1% to 6.9% among ninth-grade students, from 5.0% to 6.2% among eleventh-grade/college entry students, and from 19.1% to 24.2% among university students.

### Comparison of meningococcal detection and grouping methods.

**FIG 3:**
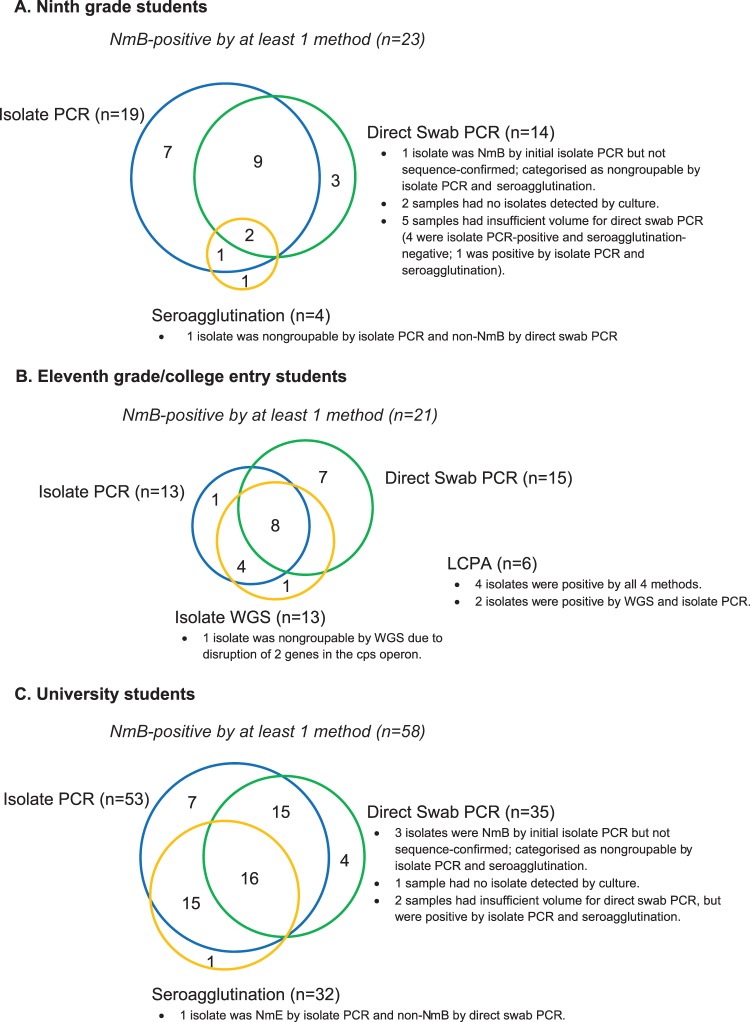
Frequency of Neisseria meningitidis serogroup B (NmB) results by 3 diagnostic methods for both cohorts. The numbers of instances in which NmB was detected for subjects by seroagglutination, isolate PCR, direct swab PCR (ninth-grade and university students); by isolate whole-genome sequencing (WGS), isolate PCR, direct swab PCR, and live cell phenotypic assay (LCPA) (eleventh-grade/college entry students); and by multiple methods are shown as Venn diagrams for ninth-grade students (A), eleventh-grade/college entry students (B), and university students (C).

NmB carriage rates determined by seroagglutination, isolate PCR analyses, and direct swab PCR were 0.8%, 1.9%, and 1.9% in ninth-grade students and 4.7%, 6.9%, and 6.1% in university students at any visit, respectively (Fig. 2A and C). NmB carriage rates among eleventh-grade/college entry students as determined by whole-genome sequencing (WGS), isolate PCR, direct swab PCR, and live cell phenotypic assay (LCPA) were 1.9%, 1.7%, 3.3%, and 0.8% at any visit, respectively (Fig. 2B). For cohorts 1 (ninth grade) and 2 (university students), NmB detection was highest by isolate PCR, followed by direct swab PCR and seroagglutination (Fig. 3). Cross-sensitivity analysis showed in each age group that direct swab PCR detected ≥50% of NmB isolates detected by the other methodologies, and isolate PCR detected ≥75% of NmB isolates detected by the other methodologies. Only 2 of 23 NmB isolates from ninth-grade students and 16 of 58 NmB isolates from university students were detected by all 3 methodologies. Because isolate PCR provided a more sensitive method for determining meningococcal serogroup among carriage isolates than did seroagglutination, rates of nongroupable isolates at any visit were higher by seroagglutination than by isolate PCR (Table 2). These results are not unexpected, given that seroagglutination is a phenotypic rather than a genotypic assay.

**TABLE 2:** Carriage rates for nongroupable isolates at any visit

Method	No. of subjects with positive non-NmB meningococcal carriage at any visit/no. ofsubjects with ≥1 culture performed at any visit (% [95% CI^*a*^]) by cohort:	
	Ninth-grade students	University students
Seroagglutination	36/533 (6.8 [4.8, 9.2])	79/360 (21.9 [17.8, 26.6])
Isolate PCR	21/533 (3.9 [2.5, 6.0])	44/360 (12.2 [9.0, 16.1])
*P* value^*b*^	0.001	<0.001

a 95% Clopper-Pearson 2-sided CI.

b McNemar test using exact method is used to compare the prevalence rate between PCR and culture/seroagglutination at each visit.

Among eleventh-grade/college entry students, WGS (*n* = 13), isolate PCR (*n* = 13), and direct swab PCR (*n* = 15) each detected ≥60% of the overall number of NmB isolates detected by any methodology, whereas LCPA (*n* = 6) detected <30%. Only 4 of 21 NmB isolates were detected by all 4 methodologies (Fig. 3).

### Multilocus sequence typing (MLST) and fHBP analyses of NmB and non-NmB isolates.

**FIG 4:**
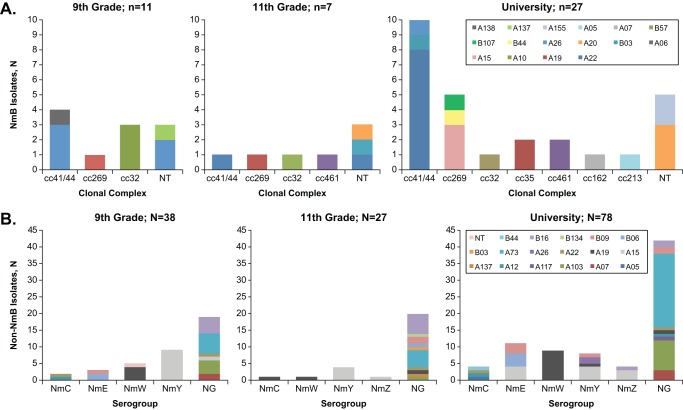
Neisseria meningitidis serogroup B (NmB) and non-NmB carriage analyses for both cohorts. NmB isolates from all 3 visits (*n* = 45) (A) were characterized by whole-genome sequencing (WGS) to determine multilocus sequence typing (MLST)/clonal complex and factor H binding protein (fHBP) assignment. Results are presented for ninth-grade, eleventh-grade/college entry, and university students. Non-NmB isolates (B) from all 3 visits (*n* = 143) were characterized by PCR (serogroup assignment, except eleventh grade by WGS) and sequence analysis (fHBP assignment). NT, nontypeable; NG, nongroupable.

Of the 48 isolates identified as NmB and subjected to whole-genome sequence analysis followed by MLST, 31.3% were typed as ST-41/44, 14.6% as ST-269, 14.6% as ST-32, and 6.3% as ST-461 complex (Fig. 4A). Nine isolates belonged to STs that have not been mapped to an existing clonal complex (CC).

At any visit, 92.3% of the 45 NmB isolates from ninth-grade students encoded fHBP subfamily A variants, as did 85.7% of isolates from the eleventh-grade/college entry students, as well as from university students (proportions of individual variants are shown in Fig. 4A). The predominant fHBP variant was A22 (33.3%); all other subfamily A variants were found in <10% of isolates with A10, A19, and A20 the next most common. Only one of the isolates (2.6%) from ninth-grade students was from fHBP subfamily B (B44), compared with 14.3% of isolates each from eleventh-grade/college entry students and the university students.

fHBP diversity was also examined in non-NmB isolates (*n* = 143), which were 76.2% subfamily A (Fig. 4B). The predominant fHBP variant was A73 (23.1%) followed by A15 (18.2%) and A19 (12.6%). B16 was the most highly represented (9.8%) subfamily B type variant, followed by B09 and B06 at 6.3% and 4.9%, respectively. Sixty-five percent of the A15 variant isolates were NmY strains, and 78% of the A19 variant isolates were NmW strains. All of the A73 variant isolates were nongroupable by the PCR assay.

## DISCUSSION

This study, focused mainly on NmB, assessed meningococcal carriage longitudinally in ninth-grade and eleventh-grade/college entry students versus university students living in residence halls and is important for understanding the epidemiology of meningococcal carriage and disease. The 2 subject groups parallel the ages preceding and corresponding to a peak in meningococcal disease incidence (6). IMD incidence among 15- to 19-year-olds in Canada was approximately 0.5 per 100,000 in 2009, with rates for NmB disease reaching nearly 0.4 per 100,000 (14). Among the same age group in Quebec in 2011, IMD incidence was 2.6/100,000 overall and 2.4/100,000 for NmB (2). In Quebec City in 2013 (last year of the study), NmB IMD incidence was 4.8 per 100,000 among those <20 years old, compared with 1.6 per 100,000 in Quebec for the same age group (15).

The study was conducted during circulation of the virulent NmB ST-269 clone (2–4), before the December 2013 approval of MenB-4C in Canada and its first use in a targeted vaccination campaign in northern Quebec in May to December 2014 (15). In this study, 29 of 45 (64.4%) NmB isolates from subjects belonged to ST-41/44, ST-269, and ST-32 CCs, which are the most frequently identified CCs among invasive isolates in Canada and Quebec (3, 14, 16).

These results are the only N. meningitidis carriage data in Quebec and may serve as baseline data for investigating NmB vaccination effects on carriage in the target age group. In the only previous study of meningococcal carriage in Canada, during a 2001 outbreak of NmC IMD in British Columbia, the overall carriage rate in persons aged 11 to 55 years was 7.6%, with a significantly lower rate in adolescents aged 11 to 12 years (1.2%) than in those aged 13 to 29 years (8.0%) (17). Higher rates in our study may be due to epidemiological, methodological, geographical, or temporal differences and/or random sampling variation.

In this study, NmB carriage rates in university students (6.9%) were higher than those in ninth-grade (1.9%) and eleventh-grade/college entry students (1.7%). This pattern is consistent with results from previous studies in the United Kingdom, where carriage prevalence among subjects aged 19 to 25 years was 6.5% (6, 18), suggesting an optimal vaccination window between ninth grade and university entry for future studies to assess prevention of meningococcal carriage and thus subsequent disease. NmB acquisition occurred relatively infrequently (<2 per 1,000 person-months for ninth-grade and eleventh-grade/college entry [13- to 18-year-old] students and 3.3 per 1,000 person-months for university [18- to 25-year-old] students), which is comparable to the rate of 2.8 per 1,000 persons-months estimated in 10- to 25-year-old students in the United Kingdom study (18).

Accurate definition of persistent carriage requires serial sampling. Persistent NmB carriage (NmB identification at ≥2 consecutive visits) was observed in 7 ninth-grade students, 5 eleventh-grade/college entry students, and 15 university students. Loss of NmB carriage was frequent, as 25% to 40% of NmB-positive students became negative for carriage during the study. Among non-NmB meningococci, NmY and NmW were most frequently detected; NmY prevalence was comparable to that of NmB in ninth-grade and eleventh-grade/college entry students but was 3 times lower (2.2%) in university students than NmB prevalence (6.9%). These results differ from the United Kingdom study reported by Jeppesen and colleagues, wherein NmY prevalence increased with age (18). Non-NmB meningococcal acquisition also occurred relatively infrequently in both cohorts, but loss of carriage was more common. Although non-NmB carriers were more common than NmB carriers, the proportion of subjects becoming negative for carriage was lower for non-NmB than for NmB. These results are important in light of an English study in which MenB-4C vaccination did not statistically alter carriage prevalence of disease-associated NmB sequence types (ST-41/44, 32, and 269) in vaccinated university students 1 month after dose 2 (19). Administration of dose 1 took place across a 3-month enrollment period; because carriage acquisition was highest between the first 2 visits, vaccination may have occurred too late to observe the greatest effects on carriage.

Classical NmB identification methodology includes seroagglutination, which was the gold standard at the time of study design, and PCR of cultured isolates, which may be preferred based on demonstrated greater sensitivity in the current study. Direct swab PCR analysis, which does not depend on successful isolate culturing, was included to determine whether this technique may be a more efficient alternative to culture-based methods. For ninth-grade students, NmB carriage rates were similar for isolate and direct swab PCR, but for university students, isolate PCR was more sensitive. Differences between these methods may be attributed to the enrichment step associated with isolate culturing. Direct swab PCR may offer little benefit in most diagnostic settings because the isolate is not available for repeat testing but may be useful when culturing at the source is not feasible.

Isolate PCR also identified fewer isolates as nongroupable meningococci than did seroagglutination, likely due to some isolates not expressing capsule. Moreover, seroagglutination assays are somewhat subjective because of reliance on visual inspection of agglutination intensity by an operator. In addition, seroagglutination reagent usage is not standardized, and reagent availability may be inconsistent. Seroagglutination is useful for characterization of invasive isolates, which most often express capsule, but may be less effective for serogrouping carriage isolates, which do not express capsule as frequently (20).

This study is the first to report fHBP variants in NmB carriage isolates in healthy subjects in Canada. In the United States, fHBP variants from subfamily A are most frequently associated with carriage in healthy adolescents and young adults, regardless of capsule locus, whereas subfamily B variants cause invasive disease most frequently in this age group (21, 22). In Canada, subfamily B variants generally cause most invasive disease in all age groups except infants (3). However, differences occur by province, with an overall predominance (including infants) of subfamily A in Ontario and subfamily B (except a predominance of subfamily A in infants) in Quebec (R. S. W. Tsang, F. B. Jamieson, B. Lefebvre, R. Gilca, S. Deeks, P. De Wals, P. Rawte, C. Tremblay, D. Law, J. Zhou, and S. Deng, 7th Vaccine and ISV Congress, poster P042, 2013). The large majority of carriage isolates in our study contained fHBP from subfamily A (A22), which is consistent with results for adolescents in the United Kingdom in which nearly 90% of NmB isolates had subfamily A variants. As carriage is considered an immunizing event, the predominance of subfamily A strains in carriage may reduce subfamily A disease in immunocompetent populations and yet result in more disease in susceptible populations such as infants.

A limitation of this study is the homogeneous study population, which should be noted when considering applicability of results to other populations. Moreover, the reported carriage rate in Quebec City may not be representative of other regions of Quebec and Canada, as has been shown for IMD isolates. Also, the population of university students in dormitories may not be representative of other populations of young adults in nonuniversity settings. In addition, the number of NmB carriers was relatively small and visits were widely spaced, preventing detection of short-term carriers. However, our study represents the largest longitudinal data set on meningococcal carriage in Canada and suggests that carriage can persist for several months, which is consistent with previous reports that 25% to 45% of carriers are persistent carriers for at least 5 to 6 months (23–25) and that 90% of persistent carriers retain the same meningococcal clone for 5 to 6 months (26).

### Conclusions.

This study informs the design of future studies assessing the effect of NmB vaccination on meningococcal carriage prevalence, its potential effect on herd immunity, and subsequent impact on IMD incidence. In this study, WGS of culture isolates detected more NmB than seroagglutination, isolate PCR, or direct throat swab PCR assays, suggesting the potential for a new standard for detecting N. meningitidis in future investigations of throat carriage. Additional research is also needed to better understand the significance of differences in distribution of NmB fHBP subfamilies and variants in carriage.

## MATERIALS AND METHODS

### Study design.

This longitudinal epidemiology study was conducted at Centre Hospitalier Universitaire (CHU) de Québec in Quebec City, Canada, between November 2010 and February 2012, with a follow-up between February 2013 and December 2013. The study was approved by the Institutional Review Board of CHU de Québec. Written informed consent was obtained from each subject or a legally acceptable representative. Between November 2010 and February 2012, enrolled subjects completed 3 office visits, followed by 3 follow-up office visits for a subset of subjects between February and December 2013 (Fig. 1).

### Study subjects.

Participants were recruited among students attending ninth-grade classes in secondary schools (cohort 1; 13 to 15 years of age at enrollment) and among those living in dormitories at universities (cohort 2; 18 to 25 years of age at enrollment) in Quebec City. Additional follow-up was conducted for a subset of cohort 1 subjects at eleventh-grade entry (16 to 18 years of age).

### Study objectives.

The primary objective was to estimate NmB throat carriage prevalence in ninth-grade students, in the same students in eleventh-grade/at college entry, and in university students living in dormitories by throat swab culture and real-time PCR-based analysis of cultured isolates at 3 time points across 5 to 8 months. Samples from eleventh-grade/college entry students were analyzed by WGS.

### Procedures.

Ninth-grade students were enrolled at 2 separate periods. Those enrolled during the first period (November to December 2010) had 3 visits, at enrollment on day 1 and approximately 3 and 6 months later; additional subjects enrolled during the second period (January to March 2011) had 2 visits. University students visited at enrollment on day 1 and approximately 6 and 20 weeks later (Fig. 1). Subjects participated for approximately 6 months. Ninth-grade students were invited to participate in a follow-up study at eleventh-grade entry. Eleventh-grade students visited at enrollment on day 1 and approximately 3 and 8 months (at college entry) later.

Two throat swabs were collected simultaneously at each office visit. One swab (culture swab) was cultured for detection and identification of *Neisseria* species at the CHU de Québec laboratory. Isolates were characterized using WGS or PCR (isolate PCR) to identify common meningococcal epidemiological markers (described in detail below) and serogrouped by standard seroagglutination testing (27). The second swab was placed in Digene specimen transport medium (Qiagen, Germantown, MD; not cultured) for direct PCR-based detection of *Neisseria.* Direct PCR analysis of storage solution from uncultured swabs and isolate PCR was conducted at a central laboratory (Pharmaceutical Product Development, LLC, Wayne, PA). Isolate WGS, MLST, and LCPA analyses were conducted at Pfizer (Pearl River, NY). Seroagglutination assays were conducted at the CHU de Québec laboratory (Table 3).

**TABLE 3:** Microbiologic analyses of samples by cohort and time point (ITT population)^*c*^

Visit (swab)	Cohort	Age group	Analysis method, *n*^*a*^					
			Seroagglutination	Isolate PCR	Direct swab PCR	Isolate WGS	Isolate LCPA	MLST
Visit 1	1	9th grade	36	36	469			
		11th grade		21	361	21	21	21
	2	University	87	102	327			
Visit 2	1	9th grade	28	28	339			
		11th grade		18	363	18	18	18
	2	University	68	77	342			
Visit 3	1	9th grade	33	34	451			
		College entry		22	356	22	22	22
	2	University	35	39	338			
Any visit^*b*^	1	9th grade	48	533	531			
		College entry		31	363	31	31	31
	2	University	99	360	359			

a *n* = number of subjects with specified test performed at that visit.

b *n* = number of subjects with ≥1 specified test performed at any visit.

c Abbreviations: ITT, intent-to-treat; LCPA, live cell phenotypic assay; MLST, multilocus sequence typing; WGS, whole-genome sequencing.

### Microbiological analysis.

Specimens were collected by simultaneously swabbing the tonsils or tonsillar fossa and posterior pharynx. Immediately afterwards, the culture swab was plated directly onto Thayer-Martin improved medium. Within 5 h, the plates were transferred to an incubator (35°C, 5% CO_2_) and monitored for up to 72 h. Colonies suspected to be N. meningitidis were subcultured on blood agar. Suspected single N. meningitidis colonies were identified as *Neisseria* species by oxidase testing, Gram staining, and biochemical identification (28) using the API NH kit (bioMérieux, St Laurent, QC, Canada). Serogrouping was performed by slide-agglutination as described previously (27).

### PCR analysis.

Real-time PCR assays (29) were conducted using TaqMan primer sets (Life Technologies, Burlington, ON, Canada) for each of the 8 capsule-specific genes of interest for N. meningitidis (NmA, NmB, NmC, NmE, NmW, NmX, NmY, and NmZ). PCR assays were additionally qualified for *porA* and *ctrA*. Based on PCR results from swab culture isolates (isolate PCR) and direct swab PCR, samples were grouped into 5 categories: all meningococci, grouped meningococci, nongroupable meningococci, non-NmB meningococci, and group B meningococci (Table 4). Direct swab PCR samples were genogrouped only for NmB. Standard PCR amplification and sequencing of the fHBP gene were also performed for NmB isolates as previously described (30).

**TABLE 4:** Definitions of meningococcal categories used in PCR analyses^*a*^

Category	Isolate PCR	Direct swab PCR	WGS
All meningococci	Any sample in which the *porA* and/or *ctrA* gene(s) was detected	Any sample in which the *porA* and/or *ctrA* gene(s) and/or the group B capsule gene was detected	Any sample in which the *porA* and/or *ctrA* sequences were predicted to encode full-length gene product
Grouped meningococci	Any sample in which the *porA* and/or *ctrA* gene(s) and the relevant group capsule gene were detected	Performed only for NmB	Any sample in which the *porA* and/or *ctrA* sequences and all the required genes for synthesis and transport of the relevant capsule group were predicted to encode full-length gene products
Nongroupable meningococci	Any sample in which the *porA* and/or *ctrA* gene(s) was detected but no capsule gene was detected	Not applicable	Any sample in which the *porA* and *ctrA* sequences were predicted to encode full-length gene product and lacking any of the required genes for synthesis and transport of the relevant capsule group OR any sample in which the *ctrA* sequence is incomplete
Non-NmB meningococci	Not applicable	Any sample in which the *porA* and/or *ctrA* gene(s) was detected but the group B capsule gene was not detected	Not applicable
Group B meningococci	See “Grouped meningococci” above	Any sample in which the group B capsule gene was detected	See “Grouped meningococci” above

a Abbreviations: NmB, *Neisseria meningitidis* serogroup B; WGS, whole-genome sequencing.

### Additional genotypic and phenotypic analyses.

Characterization of the follow-up cohort meningococcal isolates (eleventh-grade/college entry) was performed by WGS. In total, 45 unique NmB isolates, representing all 3 cohorts, were further characterized by WGS and LCPA. Detailed information about WGS and LCPA analyses is provided elsewhere (20); briefly, LCPA analyses were used to determine meningococcal serogroup by bioluminescent detection of serogroup-specific monoclonal antibody binding to isolates. MLST data were obtained as previously described (31).

### Statistical analysis.

Based on CI estimates for various prevalence rates and sample sizes, the target enrollment was a minimum of 500 to 750 ninth-grade students and 200 to 350 university students. A convenience sample size of 360 eleventh-grade students from the former ninth-grade students was selected *a priori*.

The intent-to-treat population (all enrolled subjects) was used for epidemiologic endpoint analyses. Confidence intervals were calculated using the exact method based on Clopper-Pearson (2-sided). The McNemar test using the exact method was used to compare the prevalence rates between PCR analyses and seroagglutination for each visit in ninth-grade/university students. The NmB acquisition rate was defined as the number of any new NmB carriage cases in the population in a given period of time. The rate was calculated by dividing the number of new carriage cases over time by the sum of the person-time (person-time was calculated as the sum of all initial negation subjects’ duration in the study). Data from isolate PCR analyses were used to determine acquisition rates for ninth-grade and university students, and data from WGS analysis were used to determine rates for eleventh-grade/college entry students.
